# Ecological Risk Assessment of Ammonia Nitrogen in China’s Surface Water: Implications for Environmental Management from Concentration-Risk Misalignment

**DOI:** 10.3390/toxics14070576

**Published:** 2026-06-30

**Authors:** Yue Lu, Yizhang Zhang, Guanglei Zhao, Huiling Zhang, Zhenguang Yan

**Affiliations:** 1 State Key Laboratory of Environmental Criteria and Risk Assessment, Chinese Research Academy of Environmental Sciences, Beijing 100012, China; qxly6427@126.com; 2 Research Institute for Environmental Innovation (Tianjin Binhai), Tianjin 300450, China; zhangyz_hky@163.com (Y.Z.); zglei21@163.com (G.Z.); 3 The Bureau of Urban Management and Comprehensive Law Enforcement of Xingtai, Xingtai 054000, China; 4 Southern Marine Science and Engineering Guangdong Laboratory (Zhuhai), Zhuhai 519000, China

**Keywords:** total ammonia nitrogen (TAN), ecological risk assessment, dynamic water quality criteria, water quality management

## Abstract

Total ammonia nitrogen (TAN) is a ubiquitous and critical pollutant in global surface waters. In China, regulatory oversight largely relies on static standard limits, often overlooking the influence of environmental factors on ammonia toxicity. Based on large-scale monitoring data from seven major river basins across China from 2021 to 2024, this study employed *p*H- and temperature-dependent Local Water Quality Criteria (*LWQC*) to identify the spatiotemporal decoupling between TAN concentrations and Risk Quotients (*RQ*s). The results reveal a “double-peak” seasonal pattern in TAN concentrations nationwide, namely a primary peak in winter (December to February) and a secondary peak in summer (June to August), driven by low flow during the dry season and rainfall-induced non-point source runoff, respectively. Crucially, the study confirms a significant “concentration-risk paradox”: while TAN concentrations are highest in winter, ecological risk remains at an annual low due to the protective effect of low temperatures on toxicity. Conversely, despite lower total concentrations in summer, high temperatures and elevated *p*H trigger a sharp decline in *LWQC* and a surge in the proportion of highly toxic un-ionized ammonia (NH_3_), marking summer as the peak period for ecological risk. Comparative analysis indicates that approximately 61.43% of river sections meeting the current Grade III water quality standards remain in a high-risk state. This underscores the inadequacy of static standards in providing sufficient protection during sensitive seasons. We suggest that water environmental management should shift from “concentration-based compliance” to “risk-based management,” implementing differentiated TAN control strategies specifically targeting the sensitive summer window.

## 1. Introduction

Total ammonia nitrogen (TAN) is widely distributed in global surface waters, and its biological toxicity poses a direct threat to aquatic biodiversity and ecosystem integrity [1,2]. Human activities have exerted a profound impact on the global nitrogen cycle, resulting in diverse sources of TAN in water bodies, primarily including industrial effluents, agricultural runoff, domestic sewage [3], and aquaculture wastewater [4]. In China, rapid population growth and economic development have led to a surge in anthropogenic nitrogen inputs [5], placing many river basins under severe pressure from TAN pollution. Consequently, TAN has become one of the primary target pollutants for regulatory control in China’s surface waters. Although stringent source-control measures have resulted in a significant decline in TAN concentrations nationwide, the ecological risks associated with ammonia remain a critical concern due to the complexity of its toxic effects [6].

In aquatic environments, TAN exists in a dynamic equilibrium between un-ionized ammonia (NH_3_) and ionized ammonium (NH_4_^+^) [7]. Among these forms, NH_3_ exhibits acute toxicity due to its neutral charge and lipid solubility [8], which allow it to penetrate biological membranes more readily than the ionized form [9]. This chemical equilibrium is strongly driven by the *p*H and temperature (*T*) of the water body; specifically, the proportion of NH_3_ increases exponentially with rising *p*H or *T* [10]. Consequently, the same concentration of TAN can exert vastly different biotoxic effects under varying physicochemical backgrounds. Ammonia nitrogen exhibits toxic effects on aquatic organisms, including invertebrates [11], fish [12], amphibians [13], and algae [14,15]. Given this toxicological mechanism, it is imperative to incorporate the influences of *T* and *p*H when establishing water quality criteria (WQC) for ammonia. However, China’s current Environmental Quality Standards for Surface Water (GB 3838-2002) [16] primarily categorize water quality into five classes based on functional zoning, with TAN limits set at ≤0.15, 0.5, 1.0, 1.5, and 2.0 mg/L for Grades I through V, respectively. These static standards fail to account for the toxicity mechanisms mediated by *p*H and *T*. In contrast, many countries and regions, such as the United States [17], Canada [18], and New Zealand [19], have established ammonia criteria for the protection of aquatic life based on the relationship between toxicity and environmental factors. In April 2020, China also released the Water Quality Criteria for Freshwater Aquatic Organisms—Ammonia Nitrogen, which stipulates 144 short-term and long-term WQC values tailored to different water quality conditions [20]. This development provides a robust scientific foundation for assessing the ecological risks of ammonia in China’s surface waters.

In recent years, several studies have conducted localized risk assessments based on water quality criteria in specific basins, such as the Shaying River [7], the Yellow River [21], the Liao River [22], and Chagan Lake [2]. However, most existing research has focused on single basins or short-term scales, lacking cross-climatic zone comparative studies at the national scale that utilize extensive monitoring data. Particularly at the basin scale, it remains unclear whether a spatiotemporal misalignment exists between static concentration monitoring and dynamic ecological risks, as well as the quantitative contribution of environmental factors driving such a mismatch. This lack of clarity significantly constrains the transition of China’s water environmental policy from “concentration-based target management” to “ecological risk-based management.”

In light of these research gaps, this study systematically evaluated TAN using surface water monitoring data from seven major river basins in China from 2021 to 2024. By employing the Water Quality Criteria for Freshwater Aquatic Organisms—Ammonia Nitrogen to construct a dynamic criteria model, combined with the Risk Quotient (*RQ*) method, this research aims to undertake the following:

(1) Characterize the spatiotemporal differentiation of TAN concentrations and associated ecological risks across China’s primary basins;
(2) Identify “potential risk zones” and their distribution patterns even under conditions of compliance with static water quality standards;
(3) Elucidate the driving mechanisms of *T* and *p*H on the evolution of ammonia-related risks.

## 2. Materials and Methods

### 2.1. Overview of the Study Area

China possesses an expansive network of river systems, the most significant of which are the “Seven Major River Basins”—namely the Yangtze, Yellow, Pearl, Songhua, Liao, Hai, and Huai Rivers. All of these belong to the Pacific drainage system, covering a total drainage area that accounts for approximately 47.3% of China’s landmass. These basins span diverse climatic zones, including temperate, warm-temperate, subtropical, and tropical regions. Economically and socially, the seven major basins contribute over 80% of the national Gross Domestic Product (GDP) and support more than 80% of the population, playing a pivotal role in China’s water resource management and ecological security [23].

### 2.2. Water Quality Monitoring Data

The monitoring sections in this study are national control sections, covering the main stems and major tributaries of China’s seven major river basins (Yangtze, Yellow, Pearl, Songhua, Liao, Hai, and Huai Rivers), as well as key water functional zones nationwide, ensuring robust spatial representativeness. The data frequency covers the variation cycles of water quality indicators, providing strong temporal representativeness. The raw data were retrieved from the National Real-time Publication System for Surface Water Quality Automatic Monitoring (https://szzdjc.cnemc.cn:8070/GJZ/Business/Publish/Main.html, accessed on 26 June 2026), spanning the period from 2021 to 2024. The ammonia nitrogen concentration was determined using online automatic analyzers based on Nessler’s reagent colorimetric method or the salicylate–hypochlorite method, in accordance with national standard analytical specifications HJ 535-2009 and HJ 536-2009. All supporting monitoring laboratories responsible for sample testing and data verification hold valid China Metrology Accreditation (CMA) and China National Accreditation Service for Conformity Assessment (CNAS) accreditation covering water quality indicator analysis. To ensure data quality, invalid values resulting from instrument malfunctions or other technical anomalies were identified and removed. The final dataset incorporated into this analysis comprises a total of 118,521 valid observations.

The fraction of TAN present as un-ionized ammonia (NH_3_) was calculated using the temperature-dependent equilibrium equations of Emerson et al. [24]. The dissociation constant (*pK_a_*) was first computed as

(1) $${pK}_{a}=0.09018+\frac{2727.92}{T}$$

where *T* is the absolute temperature (K). The NH_3_ fraction (*f*) was then determined from the sample *p*H as

(2) $$f=\frac{1}{10^{{pK}_{a}-pH}+1}$$

The NH_3_ concentration (mg/L as N) was subsequently obtained by multiplying f by the measured TAN concentration. This calculation assumes freshwater conditions and is valid for temperatures between 0 and 50 °C, which encompass the range of our monitoring data.

### 2.3. Derivation of Dynamic Water Quality Criteria

The Water Quality Criteria for Freshwater Aquatic Organisms—Ammonia Nitrogen, released by China’s Ministry of Ecology and Environment (MEE) in April 2020, derived both Short-term Water Quality Criteria (SWQC) and Long-term Water Quality Criteria (*LWQC*). As shown in Table 1, a total of 144 criteria values were established across 72 different combinations of water temperature and *p*H in freshwater ecosystems. These values represent the maximum concentration of TAN that exerts no adverse effects on 95% of freshwater aquatic species and their ecological functions under specific water quality conditions. *LWQC* refers to the water quality thresholds designed to protect aquatic life and ecological functions under chronic exposure. In this study, *LWQC* refers to the water quality thresholds established to protect aquatic organisms and ecosystem functions from long-term TAN (as N) contamination. To ensure a higher level of protection for freshwater ecosystems, this study adopted the more stringent *LWQC* to evaluate and quantify ammonia-related risks. To obtain the specific criteria value for each monitoring section at a given time point, we employed the Bilinear Interpolation method. This allowed us to calculate the corresponding long-term criteria value (*LWQCi*,*t*) within the criteria matrix based on the field-measured *p*H and T data.

The calculation is expressed as follows:

(3) $${\mathrm{LWQC}}_{i,t}\;=\;f\left({pH}_{i,t},{\;T}_{i,t}\right)$$

where *LWQCi*,*t* represents the dynamic long-term water quality criteria (mg/L) for the *i*-th monitoring section at time *t*. This approach ensures that the criteria values reflect, in real time, the influence of evolving physicochemical environments on biological sensitivity. Compared to traditional static assessments, this method offers greater biological relevance by accounting for the fluctuating toxicity of ammonia.

### 2.4. Ecological Risk Assessment Method

The *RQ* method is widely utilized in ecological risk assessments due to its relative simplicity, effectiveness, and interpretability [25]. In this study, the *RQ* method was employed to evaluate the potential pressure of TAN on aquatic ecosystems. The *RQ* value is calculated as the ratio of the measured environmental concentration to the water quality criteria, expressed as follows:

(4) $$\mathrm{RQ}\;=\;\frac{{\mathrm{MEC}}_{i,t}}{{\mathrm{LWQC}}_{i,t}}$$

where *MECi*,*t* represents the Measured Environmental Concentration (mg/L) of TAN for the *i*-th section at time t. Following the classical risk classification standards [26,27], the ecological risk levels of ammonia nitrogen were categorized into three tiers based on the *RQ* values:

(1) *RQ* < 0.1: Low ecological risk;
(2) 0.1 < *RQ* ≤ 1: Moderate ecological risk;
(3) *RQ* > 1: High ecological risk, indicating that the concentration has exceeded the biological protection threshold.

Statistical comparisons among multiple groups (river basins and seasons) were performed using one-way analysis of variance (ANOVA), followed by Tukey’s Honest Significant Difference (HSD) post hoc test for pairwise multiple comparisons. The family-wise error rate was controlled at α = 0.05 using this adjustment.

### 2.5. Correlation Analysis Methods

Since the relationships among *T*, *p*H, *LWQC*, TAN concentration, and *RQ* are not strictly linear and do not conform to a normal distribution, this study employed Spearman’s rank correlation coefficient (rs) for correlation analysis to identify the primary drivers of ammonia risk.

Proposed by Charles Spearman, the Spearman rank correlation coefficient is a non-parametric (distribution-free) rank statistic used as a measure of the strength of association between two variables [28]. As a scale-invariant measure of consistency, rs is particularly suitable for analyzing variables with unknown distributions or potentially non-linear correlations [21]. The value of rs ranges from [−1, 1], where a larger absolute value indicates a stronger correlation. When rs = 1, the monotonic relationship between the two variables is perfectly consistent; when rs = −1, the monotonic relationship is perfectly opposite. The correlation strength was categorized based on the absolute value of rs (|rs|):

(1) |rs| ≥ 0.7: Strong correlation;
(2) 0.4 ≤ |rs| < 0.7: Moderate correlation;
(3) 0.2 ≤ |rs| < 0.4: Weak correlation;
(4) |rs| < 0.2: Very weak or no correlation.

## 3. Results

### 3.1. Spatiotemporal Distribution Characteristics of TAN Concentrations

**Figure 1:**
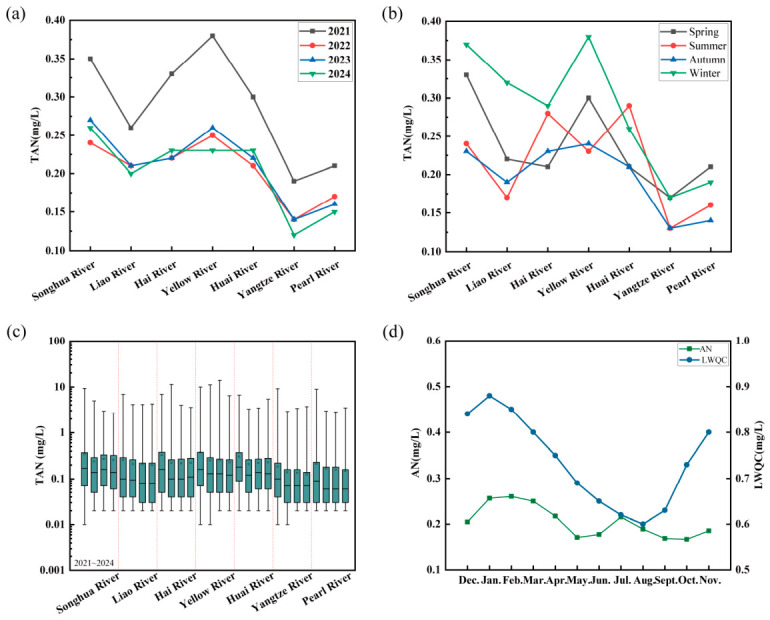
Spatiotemporal characteristics of ammonia concentrations across seven major river basins from 2021 to 2024: (**a**) annual mean concentrations; (**b**) seasonal variations in concentration; (**c**) annual boxplots of ammonia levels; (**d**) monthly dynamics of Total ammonia nitrogen (TAN) concentrations versus *LWQC*.

Monitoring data from 2021 to 2024 reveal a significant “high in the north, low in the south” spatial pattern for TAN concentrations in China’s surface waters (Figure 1a). During this period, the annual mean TAN concentrations across the seven major river basins fluctuated between 0.18 and 0.25 mg/L. Among these, the Yellow, Songhua, and Hai River basins exhibited relatively high TAN loads, with annual mean concentrations in certain river sections exceeding 0.25 mg/L. This trend is primarily attributed to the limited hydraulic dilution capacity caused by lower river discharge in northern China, coupled with intensive anthropogenic activities such as agricultural non-point source pollution and domestic sewage discharge. In contrast, the Yangtze and Pearl River basins generally maintained lower concentration levels, typically falling within the Grade I–II limits of the GB 3838-2002 standard (annual mean concentrations ranging from 0.12 to 0.21 mg/L). This is largely due to the abundant precipitation and sufficient runoff replenishment characteristic of southern river systems.

From a temporal perspective, TAN concentrations across all basins exhibited highly synchronized seasonal patterns. Notably, rather than following a simple linear trend, TAN concentrations at the national scale displayed a distinctive “double-peak” characteristic (Figure 1d). In addition to the primary peak in winter—likely caused by reduced water volume during the dry season and the inhibition of nitrification by low temperatures—a conspicuous secondary peak was observed during the summer months (June–August).

During the 2021–2024 study period, the overall TAN concentrations across China’s seven major river basins showed a steady decline or remained at low-level fluctuations (Figure 1a). This trend reflects the phased achievements of the “targeted pollution control” policies implemented during China’s 14th Five-Year Plan period. However, despite the significant reduction in physical concentrations, localized concentration anomalies persisted in the arid and semi-arid basins of northern China during winter, posing a potential environmental pressure.

**Figure 2:**
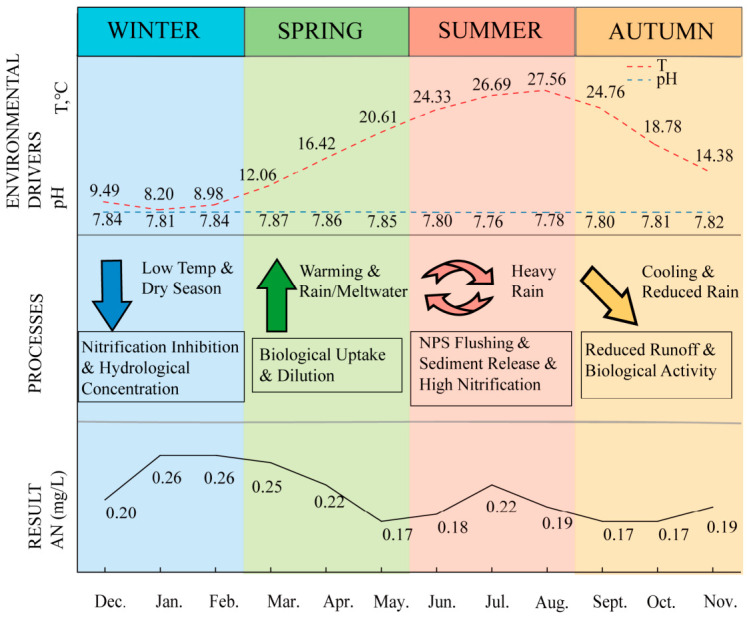
A conceptual scheme of seasonal changes in environmental conditions and processes affecting TAN.

The water quality criteria for ammonia are heavily dictated by water temperature and *p*H, leading to pronounced and regular seasonal fluctuations in the *LWQC* across the seven major river basins (Figure 1d). In contrast to the “double-peak” trend observed in TAN concentrations, the *LWQC* exhibited a distinctive “single-trough” pattern. In winter, the *LWQC* remained at its annual peak (mean: 0.84 mg/L). This is because the low-temperature environment significantly reduces the proportion of NH_3_, thereby enhancing the tolerance of aquatic organisms to TAN and creating a wide safety buffer (Figure 2). Conversely, the *LWQC* experienced a sharp decline in summer, with a mean of only 0.63 mg/L, and in some periods, it dropped to merely 1/5 to 1/10 of the winter criteria values. This plummet is synergistically driven by rising temperatures and upward shifts in *p*H, implying that even at lower TAN concentrations, aquatic ecosystems remain in an extremely sensitive state during summer. While the Measured Environmental Concentration (MEC) reaches its primary peak in winter, the *LWQC* is simultaneously at its highest level. The resulting large margin between the two leads to relatively low *RQ* values. In summer, however, although the MEC only reaches a secondary peak (annual mean ~0.19 mg/L), the *LWQC* contracts to extremely low levels—at times falling below the actual concentrations measured at certain sections. Consequently, the MEC curve frequently “encroaches upon” or even penetrates the *LWQC* curve during the summer months.

### 3.2. Spatiotemporal Dynamics of Ammonia Ecological Risk

The ecological *RQ* of TAN across the seven major basins displayed a trend distinct from the TAN concentration curves. Although TAN concentrations in summer remained at a secondary peak or even relatively low levels, the extreme tightening of the *LWQC*—driven by elevated *p*H and water temperature—resulted in a spatiotemporal coupling and synergistic effect. This synergy led to a substantial increase in ecological risk, with the national average *RQ* peaking in July (mean: 0.52). During this period, the proportion of river sections classified as high risk (*RQ* > 1.0) surged from 8.46% in winter to 11.60%. Conversely, while TAN concentrations were highest in winter, the high-level protection afforded by the *LWQC* kept the *RQ* values at a lower level (mean: 0.37), with the majority of sections (52.28%) exhibiting moderate risk (0.1 < *RQ* ≤ 1).

**Figure 3:**
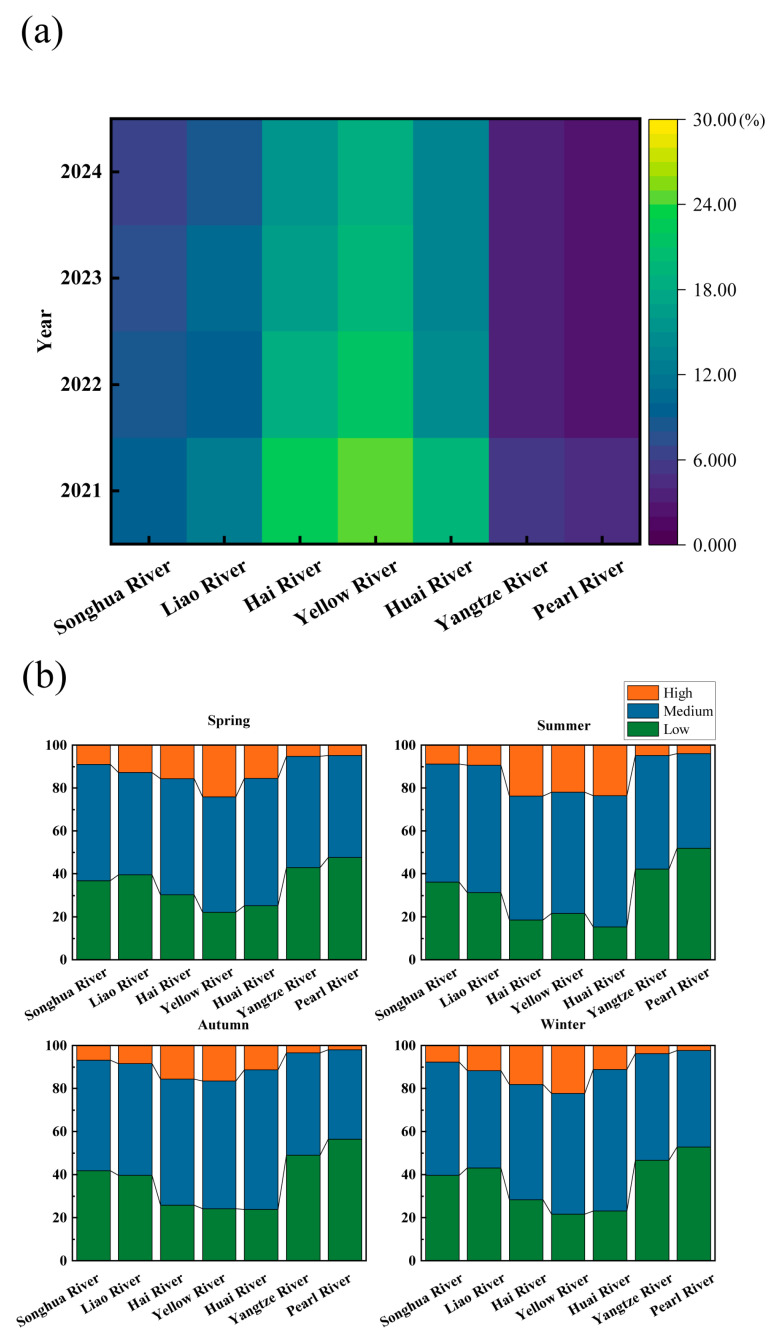
Ecological risks of ammonia across seven major river basins from 2021 to 2024: (**a**) heatmap of ammonia ecological risk; (**b**) seasonal variations in ecological risk levels.

The regions with high ammonia risk were primarily concentrated in northern China (Figure 3a), particularly in the Yellow, Hai, and Huai River basins. The proportions of high-risk sites in these basins were significantly higher than in the other four basins, with annual mean proportions of 21.10%, 18.27%, and 15.47% (Figure 3b), respectively. In these three basins, summer *p*H levels remained high (mean > 7.8), and TAN concentrations rebounded due to summer non-point source runoff, resulting in a significantly higher proportion of high-risk ammonia nitrogen *RQ* points compared to other river basins (*p* < 0.05). The TAN concentrations driving this high-risk pattern are likely derived from intensive anthropogenic nitrogen emissions from agricultural, industrial, and residential sources within the basins. In contrast, the Yangtze and Pearl River basins exhibited minimal *RQ* fluctuations throughout the year, with high-risk sites accounting for less than 5% annually. This is attributed to their abundant water volume, strong dilution capacity, and relatively neutral *p*H levels, with moderate risk appearing only seasonally in localized tributaries.

### 3.3. Discrepancies Between Static and Dynamic Standard Assessments

**Figure 4:**
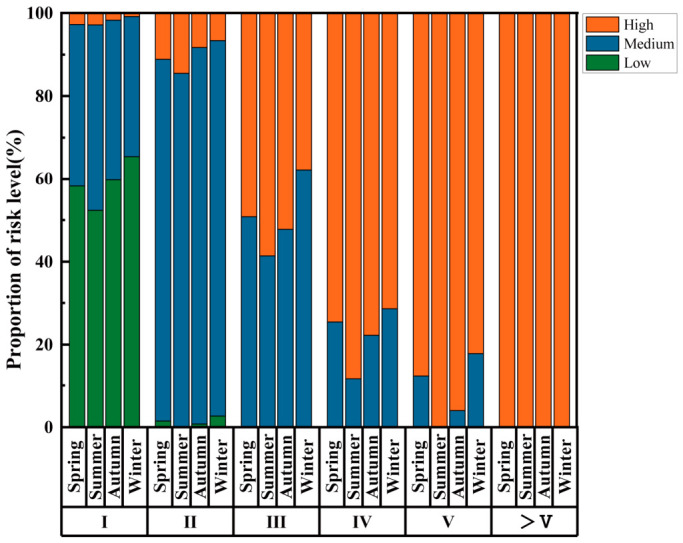
Seasonal risk distribution (high, moderate, and low) for river sections meeting GB 3838-2002 water quality standards.

An evaluation of TAN concentrations across the seven major river basins from 2021 to 2024, based on the GB 3838-2002 standards, reveals that the static Grade III water quality limit fails to identify 61.43% of the river sections categorized as moderate-to-high risk by the *LWQC*-based dynamic assessment. As illustrated in Figure 4, a significant portion of the river sections categorized as Grade I and II water quality (based on GB 3838-2002) still exhibited low, moderate, and high ecological risks. Despite the extremely low TAN concentrations, approximately 56.56% of these sections remained at moderate risk (0.1 < *RQ* ≤ 1), and 6.29% even triggered high-risk warnings (*RQ* > 1) under the high-temperature and high-*p*H conditions prevalent in summer. In sections classified as Grade III to V, low-risk instances vanished entirely, leaving only moderate and high risks. The Grade III interval exhibited the most severe misalignment; among all Grade III samples, the proportion of high-risk cases reached as high as 48.67%. This indicates that the current static threshold of 1.0 mg/L is excessively lenient for protecting alkaline water bodies in summer (such as those in the Yellow River Basin), failing to account for the dynamic surge in ammonia toxicity.

In summer, while TAN concentrations in most river sections remain within the Grade III limit, the dynamic assessment reveals that approximately 9.59% of these “compliant” sections are, in fact, in a high-risk state. This “standard failure rate” is particularly prominent in northern alkaline waters during summer, revealing significant spatiotemporal blind spots in the current regulatory framework regarding the protection of aquatic organisms from ammonia toxicity.

### 3.4. Seasonal Dynamics of Ammonia Speciation and the Toxicity Amplification Effect

**Figure 5:**
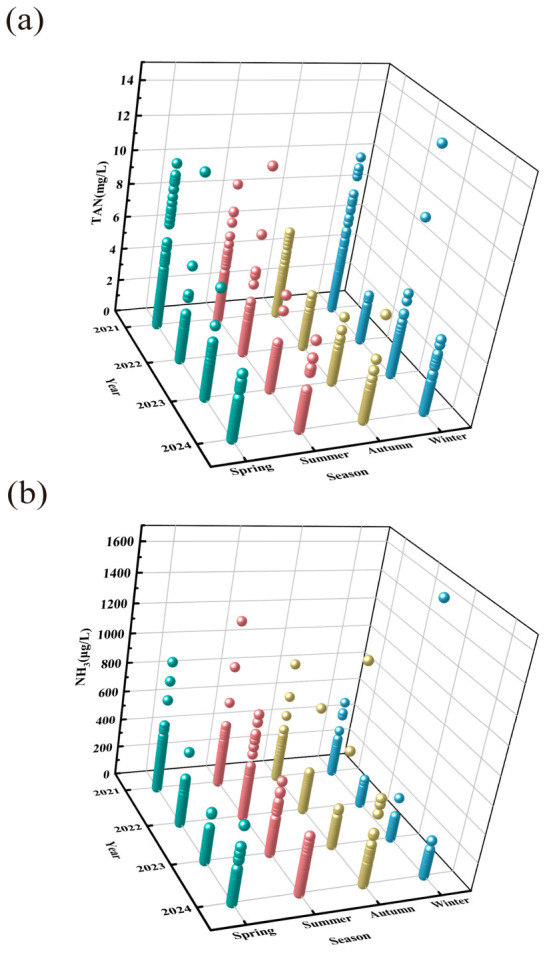
Seasonal dynamics of TAN and NH_3_ across seven major river basins from 2021 to 2024: (**a**) the seasonal variation trend of TAN; (**b**) the seasonal variation trend of NH_3_.

When comparing seasonal concentrations of TAN and NH_3_, a distinct divergence emerges. In winter, TAN concentrations reach their peak while NH_3_ concentrations fall to their annual minimum (Figure 5). This indicates that in low-temperature environments (average *T* ≤ 10 °C), even when TAN concentrations are elevated (>1.0 mg/L), the conversion to the toxic NH_3_ form is suppressed to below 15%. Under these conditions, the majority of ammonia exists as relatively non-toxic ionized ammonium (NH_4_^+^). In contrast, summer exhibits lower TAN concentrations but the highest NH_3_ levels, revealing a significant “toxicity amplification effect” synergistically driven by high temperature and elevated *p*H. Even when TAN remains at relatively low levels (0.15–0.5 mg/L), high concentrations of NH_3_ can still be generated. This leads to a rapid surge in the concentration of the un-ionized form, markedly enhancing the overall toxicity and escalating the ecological risk of ammonia.

### 3.5. Correlation Analysis

**Figure 6:**
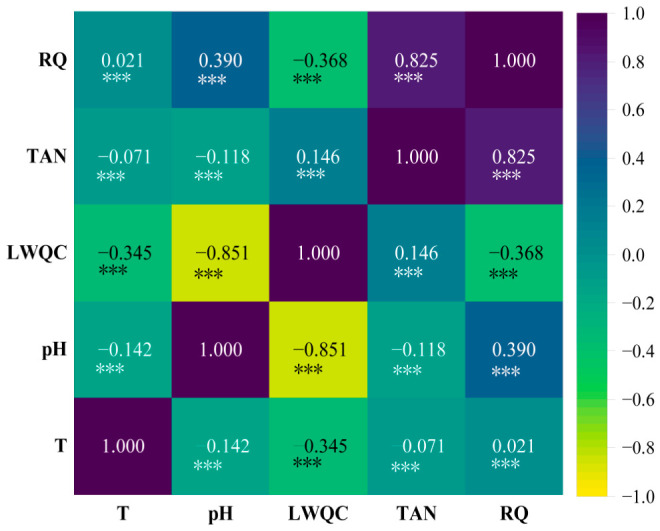
Spearman’s rank correlation analysis among water temperature (*T*), *p*H, *LWQC*, TAN concentration, and Risk Quotient (*RQ*) (*** representation *p* < 0.001).

Figure 6 presents the results of Spearman’s rank correlation analysis among *T*, *p*H, *LWQC*, TAN, and *RQ*, with a total sample size of 118,521. The ranking of TAN concentrations and *RQ* values showed high consistency, exhibiting a strong positive correlation (rs = 0.825, *p* < 0.001). In contrast, *p*H demonstrated low consistency with *RQ* rankings, showing a weak positive correlation (rs = 0.390, *p* < 0.001), while water temperature showed almost no consistency with *RQ*, resulting in a negligible positive correlation (rs = 0.021, *p* < 0.001). These findings indicate that the ecological risk of ammonia across the seven major basins is primarily governed by TAN concentrations. Furthermore, *p*H showed an inverse ranking consistency with *LWQC*, characterized by a strong negative correlation (rs = −0.851, *p* < 0.001). Water temperature exhibited a weak negative correlation with *LWQC* (rs = −0.345, *p* < 0.001), suggesting that the *LWQC* values are predominantly influenced by *p*H. Due to the inherent weak correlations among these water quality indicators, it remains challenging to accurately quantify the individual contributions of *T*, *p*H, and TAN concentration to the *RQ* using traditional models such as linear or logistic regression [21]. Traditional linear regression models are insufficient for accurately attributing the contributions of *p*H, *T*, and TAN to *RQ*, because their relationships are inherently non-linear and interactive. In contrast, recent studies have successfully applied machine learning models combined with SHAP to decompose TAN risk into the contributions of key influencing factors [29].

## 4. Discussion

### 4.1. Spatiotemporal Differentiation of Ammonia Risk

The ecological risk of ammonia exhibits pronounced spatial heterogeneity across China’s seven major river basins, reflecting regional disparities in anthropogenic pressure, hydrological characteristics, and water chemistry [30]. Our findings reveal a significant “high in the north, low in the south” spatial pattern of ammonia risk. This differentiation not only reflects differences in natural hydrological backgrounds but also profoundly underscores the divergent patterns of anthropogenic nitrogen inputs across these basins. In northern China, the high ammonia risks in the Yellow, Hai, and Huai River basins are primarily driven by excessive TAN loads. Due to low river discharge, these basins possess extremely limited hydraulic dilution and self-purification capacities, leading to the rapid accumulation of pollutants during dry seasons [31]. As a critical base for energy-intensive heavy industry and irrigated agriculture, the Yellow River basin exhibits a dual characteristic of “high-intensity emissions and low environmental capacity.” In particular, the concentration of coal chemical and non-ferrous metal smelting industries contributes to persistently high intensities of industrial ammonia discharge [32]. Conversely, in southern China, surface waters are undergoing a continuous and accelerating process of alkalization [33,34]. Although the TAN concentrations in the Yangtze and Pearl River basins remain relatively low, the high temperatures and elevated *p*H levels significantly amplify the ecological risk of ammonia, especially during the summer months [29].

The seasonal “double-peak” evolution of TAN concentrations reflects the intertwined effects of natural hydrological cycles and intense anthropogenic interference. The formation of the primary peak in winter is mainly attributed to the physical concentration of point-source emissions. During the dry season, river discharge reaches its annual minimum, preventing the effective dilution of urban domestic sewage and industrial wastewater. Furthermore, the strong inhibition of nitrifying bacteria metabolic activity by low temperatures significantly reduces the water body’s self-purification capacity [35,36,37]. The secondary peak in summer is primarily driven by rainfall-induced non-point source runoff. In agriculture-intensive basins such as the Yellow and Huai Rivers, summer coincides with both high-intensity fertilization and concentrated monsoon rainfall. Surface runoff generated by heavy rains rapidly flushes residual nitrogen fertilizers from farmlands and livestock waste into river channels, creating a massive pollutant load. This non-point source flushing effect offsets the benefits of hydraulic dilution and enhanced biodegradation during the high-flow summer season, establishing the material basis for risk outbreaks. Additionally, the endogenous release from bottom sediments triggered by high summer temperatures further exacerbates TAN accumulation. These findings underscore the critical importance of strengthening non-point source pollution control during the rainy season to mitigate basin-wide ecological risks.

The ecological risk of ammonia also exhibits a seasonal “double-peak” distribution, with high risks observed in both summer and winter, albeit driven by different causes. In northern China, the risk is primarily concentrated in winter, where high volumes of pollutant discharge and low dilution capacity lead to elevated TAN concentrations. In southern China, the risk reaches its maximum during summer, as high temperatures trigger an increase in the content of NH_3_, which enhances the toxicity of ammonia and subsequently amplifies the ecological risk. This pronounced seasonal heterogeneity underscores the demand for dynamic management.

### 4.2. Mechanism Analysis Driving the Decoupling of TAN Concentration and Risk

Traditional views generally assume that high pollutant concentrations directly correspond to high ecological risks. However, this study observed a significant “concentration-risk inversion” phenomenon across the seven major basins: while TAN concentrations are “high in winter and low in summer,” the ecological risk (*RQ*) is “high in summer and low in winter.” The core of this “concentration-risk paradox” lies in the fact that the proportion of the toxic ammonia species (NH_3_) is not linearly driven by the total amount but is instead governed by a thermodynamic equilibrium process regulated by environmental factors. High concentrations in winter are mainly controlled by microbial kinetics and hydrological processes: low temperatures inhibit the activity of nitrifying bacteria, hindering the conversion of ammonia to nitrate; simultaneously, reduced river discharge during the dry season exacerbates the concentration effect of pollutants. However, the low-temperature environment also substantially increases the tolerance thresholds of aquatic organisms to ammonia (i.e., higher *LWQC* values), thereby “shielding” the ecological risk brought by high concentrations to some extent.

Conversely, although intense nitrification and the dilution effect of the high-flow season reduce TAN concentrations to their annual minimum in summer, the synergy of high temperature and high *p*H causes the proportion of highly toxic NH_3_ to rise exponentially [24]. During this period, the *LWQC* tighten sharply, meaning even minor fluctuations in TAN concentration can trigger high-risk warnings. This indicates that in basins with significant monsoon climates, focusing solely on the reduction in total nitrogen concentrations is no longer sufficient to evade acute ecological risks in summer; the seasonal fluctuations of environmental physicochemical factors must be taken into account.

### 4.3. Intensification of Ammonia Risk Under Climate Change

According to the Blue Book on Climate Change in China (2022) released by the Climate Change Centre of the China Meteorological Administration, the warming rate in China reached 0.26 °C per decade from 1951 to 2021, which is higher than the global average warming level during the same period. Rapid warming creates significant uncertainties for the future climate [38], and the frequency and total duration of extreme high-temperature events in China have increased sharply in recent years [39]. Under the context of climate change, rising temperatures and *p*H variations [40], along with increasingly severe pollution [41], are all driving factors for the increase in ammonia risk.

The Blue Book on Climate Change in China notes that from 1961 to 2021, China’s average annual precipitation showed an increasing trend, with an average increase of 5.5 mm per decade. Precipitation plays a dual role: diluting ammonia nitrogen in certain cases, while activating and dispersing pollution in others [30]. Altered precipitation patterns triggered by climate change will intensify non-point source flushing in summer. More frequent extreme precipitation events in the future may lead to ammonia nitrogen entering water bodies in the form of “toxicity pulses” with higher concentrations and shorter time scales.

### 4.4. Misalignment Between Risk Peaks and Biological Life Cycle Windows

From the perspective of systems ecology, the most destructive characteristic of the summer risk peak lies in its high synchronization with the life cycle susceptibility of aquatic organisms. The period from June to August is the critical window for spawning and larval development for most fish and benthic species in China [42]. Due to immature gill tissue development and weak osmoregulation mechanisms [9,43], larvae exhibit far higher sensitivity to NH_3_ than adults [44]. The population recruitment of aquatic organisms relies heavily on the temporal matching of environmental events [45]. This study found that the *LWQC* is most stringent in summer, which precisely covers the vulnerable window of aquatic life cycles. This implies that even if the annual average ammonia concentration remains at a low level, instantaneous risk excursions in summer could cause severe damage to aquatic organisms and inhibit population recruitment rates. This “seasonal risk shock” may provide a partial explanation for the phenomenon where biodiversity recovery remains slow in some basins despite meeting water quality standards.

### 4.5. Implications for Water Quality Management

This study demonstrates that traditional ammonia management standards underestimate the risks posed by ammonia in surface waters. In other words, if the thermodynamic equilibrium effects of *T* and *p*H on NH_3_ are ignored, more than 60% of moderate-to-high risk river sections will be overlooked. This phenomenon highlights the limitations of current static management standards in protecting aquatic organisms, manifesting as regulatory gaps during the summer in northern alkaline basins and potential over-protection during the winter in southern basins. Against the backdrop of accelerating climate change, these underestimated risks cannot be ignored. It is imperative to develop water quality standards and management policies that are adaptive to climate change.

## 5. Conclusions

This study provides a comprehensive assessment of ammonia across seven major river basins in China, uncovering a profound spatiotemporal decoupling between mass concentrations and actual ecological risks. Our results demonstrate that while winter marks the zenith of TAN concentrations due to hydrological cryo-concentration, the true ecological risk manifests in summer. This summer risk surge is synergistically driven by a toxicity amplification effect—stemming from elevated *T* and *p*H—and intensified by rainfall-induced non-point source pulses.

Crucially, we identified a alignment between peak summer risks and the critical ontogenetic windows (spawning and early development) of aquatic biota, creating a “bottleneck” that hampers biodiversity restoration despite improving water quality metrics. These findings highlight a potential limitation of current management frameworks that are anchored in static concentration-based thresholds, as they may not sufficiently account for the dynamic toxicity of ammonia under fluctuating environmental conditions.

To bridge this gap, a paradigm shift from “concentration-oriented” to “bioavailability-based dynamic risk management” is imperative. Implementing seasonally adjusted water quality criteria and targeted load reduction strategies during high-sensitivity summer windows will be essential to bolstering the ecological resilience of aquatic systems in an era of accelerating global warming.

### Limitations of the Study

Several limitations should be acknowledged. Theoretically, our analytical framework is robust for assessing ammonia pollution risks based on national criteria. In practice, however, the current dataset—covering a four-year period (2021–2024) across seven major river basins—may have limited the capacity to capture long-term (decadal) trends or full spatial heterogeneity across all sub-basins and localized water bodies. Extended monitoring over a longer time frame and broader spatial coverage would further enhance the generalizability of our findings.

## Figures and Tables

**Table 1:** Chinese Local Water Quality Criteria (*LWQC*) of ammonia nitrogen for the protection of freshwater organisms/(mg/L).

	*p*H											
*T* (°C)	6.0	6.5	7.0	7.2	7.4	7.6	7.8	8.0	8.2	8.4	8.6	9.0
5	2.1	2.0	1.8	1.6	1.4	1.2	0.90	0.70	0.50	0.34	0.24	0.12
10	2.0	2.0	1.7	1.6	1.4	1.1	0.85	0.65	0.46	0.32	0.22	0.11
15	1.9	1.8	1.6	1.5	1.3	1.0	0.80	0.60	0.42	0.29	0.20	0.090
20	1.8	1.7	1.5	1.3	1.1	0.90	0.70	0.55	0.38	0.23	0.16	0.080
25	1.5	1.5	1.3	1.2	1.0	0.70	0.55	0.42	0.30	0.21	0.15	0.075
30	1.2	1.2	1.0	0.90	0.75	0.65	0.50	0.37	0.26	0.19	0.13	0.065

## Data Availability

The raw data supporting the conclusions of this article will be made available by the authors on request.
